# Quality Prediction and Control of Assembly and Welding Process for Ship Group Product Based on Digital Twin

**DOI:** 10.1155/2020/3758730

**Published:** 2020-10-18

**Authors:** Lei Li, Di Liu, Jinfeng Liu, Hong-gen Zhou, Jiasheng Zhou

**Affiliations:** School of Mechanical Engineering, Jiangsu University of Science and Technology, Zhenjiang 212003, China

## Abstract

In view of the problems of lagging and poor predictability for ship assembly and welding quality control, the digital twin technology is applied to realize the quality prediction and control of ship group product. Based on the analysis of internal and external quality factors, a digital twin-based quality prediction and control process was proposed. Furthermore, the digital twin model of quality prediction and control was established, including physical assembly and welding entity, virtual assembly and welding model, the quality prediction and control system, and twin data. Next, the real-time data collection based on the Internet of Things and the twin data organization based on XML were used to create a virtual-real mapping mechanism. Then, the machine learning technology is applied to predict the process quality of ship group products. Finally, a small group is taken as an example to verify the proposed method. The results show that the established prediction model can accurately evaluate the welding angular deformation of group products and also provide a new idea for the quality control of shipbuilding.

## 1. Introduction

The current ship production basically adopts the block construction mode, and the block is composed of a large number of typical products (including a large group, middle group, small group, t-type component, and chip component). These group products are the main object of current ship automation and intelligent construction. Due to the large quantity, large batch, and short construction cycle, the construction quality and efficiency are particularly important to improve the overall ship construction capacity. In the process of assembly and welding, a large number of quality data and process data will be generated, which is the key to evaluate the quality and can provide process decision-making support for subsequent quality prediction. Therefore, it is of great significance to realize the quality prediction and control of welding process by collecting and analyzing the data related to the welding process, so as to improve the quality and efficiency of ship product construction.

The prediction and control improve product quality and reduce production cost, which is an indispensable link to realize digital, automatic, and intelligent production. Many scholars have conducted extensive research on welding quality prediction and control. Öberg and Sikström used arc voltage measurement, CMOS vision, and infrared camera to online monitor the quality characteristics of weld penetration and evaluated the industrial applicability and applicability of the framework [1]. Zhang et al. developed a servo welding experimental system, which can extract the electrode indentation from the servo encoder to reflect the change of welding quality. This online detection method can accurately judge whether the weld meets the requirements of tensile shear strength [2]. Shi et al. made NiTi coating on stainless steel by TIG surfacing process to improve cavitation erosion resistance [3]. Yang et al. established a new welding inspection system based on 3D reconstruction technology. The support vector machine is used to evaluate the welding quality. The experimental results show that the system can complete the welding quality detection quickly and efficiently [4]. Huang and Kovacevic have developed a laser-based nondestructive vision system. By processing the image obtained by the visual sensor, the geometric characteristics of the weld can be obtained. The position and size of welding defects can be accurately identified according to the three-dimensional contour of weld, so as to realize the nondestructive testing of weld quality [5]. Gu et al. proposed an automatic tracking system for multipass welding of an arc welding robot. The system can accurately track the cap passes, filling passes, and the root pass [6]. Wang et al. proposed a new welding quality inspection framework, which can classify and evaluate welding quality and achieved good welding monitoring performance [7]. According to the literature analysis, online data monitoring and nondestructive quality assessment after welding are the main methods of quality control, which fail to effectively use the real-time data of the production process. The real-time and predictive quality control is poor, and it is difficult to meet the requirements of process online decision-making and future intelligent manufacturing development.

Digital twin technology was proposed by Michael Grieves in 2003 [8]. The current research and applications are mainly focused on product design, product quality analysis, and life prediction in the fields of aerospace and automobile manufacturing. As an emerging technology oriented to intelligent manufacturing, it has attracted wide attention. In 2011, NASN established the virtual twin of the space vehicle to predict the life of the physical vehicle [9]. Subsequently, NASA summarized previous studies on digital twins and proposed a technical route of “modeling-simulation-information technology processing,” which further increased the feasibility of digital twin technology [10]. Cai et al. created a “digital twin” virtual machine tool for physical manufacturing based on sensor data integration and information fusion technology [11]. Söderberg et al. proposed the concept of digital twins for real-time geometric guarantees and the process of how to move from mass production of personalized production [12]. Bilberg and Malik proposed a human-robot assembly system based on digital twin, which was used for industrial applications of variant assembly system to ensure the flexibility and automation of assembly [13]. Roy et al. and Lu et al. summarized the development achievements of digital twinning and pointed out the problems faced by the research of digital twinning of intelligent manufacturing [14, 15]. Park et al. and Leng et al. apply the digital twin technology to the personalized production to increase the flexibility of the manufacturing system [16, 17]. In China, there are more and more researches on digital twin. Fei et al. proposed the concept of digital twin shop-floor, discussed the basic theory and key technology of realizing information physical fusion of digital twin workshop, and then proposed the concept of five-dimension digital twin, which provided a reference for enterprises to practice digital twin [18–21]. Zhuang et al. further elaborated the connotation of digital twins, established the architecture of digital twins, and proposed the development direction of digital twins of products [22]. Qi et al. proposed the digital twin service for intelligent manufacturing and further elaborated the integration process of manufacturing service and digital twin [23]. Xie et al. proposed a virtual monitoring method for hydraulic supports based on digital twins, which simulates the behaviors of the actual hydraulic supports in the whole life cycle to achieve the synchronization of virtual and actual movements [24]. In the field of manufacturing, GE, Siemens, PTC, Dassault, and DNV GL use digital twin technology to meet their respective enterprise needs [25–29]. Through the above analysis, it can be seen that the digital twin technology makes full use of physical models, sensor update, and historical data and integrates multidisciplinary and multiphysical quantities, multiscale, and multiprobability. It can complete the interactive mapping of virtual and physical space, which is an effective means to achieve decision-making and quality control.

In recent years, the research team has deeply studied the dynamic evaluation method and process quality prediction technology of complex product processing technology based on digital twin [30–32], which has improved the timeliness and effectiveness of machining technology evaluation and quality prediction. On the basis of preliminary research, a quality prediction and control method for assembling and welding of group products based on digital twin is proposed. Firstly, a digital twinning model of welding quality prediction and control including physical assembly and welding entity, virtual assembly and welding model, welding quality prediction and control system, and twinning data was established. Then, by means of real-time data collection based on the Internet of Things, twin data organization based on XML, and process quality prediction based on machine learning, the quality control of assembly and welding process of ship group products are realized. Lastly, a small group product of ship is taken as objects for verification.

## 2. The Quality Factors of Assembly and Welding for Ship Group Products

**Figure 1:**
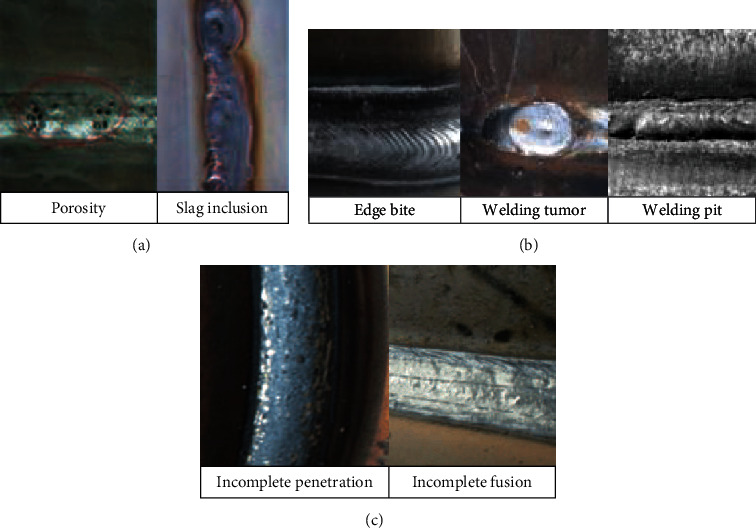
Defects of ship surface welding quality: (a) bulk defects; (b) surface defects; (c) linear defects.

The assembly and welding process of ship group products mainly include four steps: marking, spot welding, welding, and quality inspection. Hull welding involves physics, chemistry, metal material science, and welding metallurgy. The main defects of the ship welding quality include bulk defects (porosity, slag inclusion, etc.), surface defects (bite edge, welding tumor, welding pit, etc.), and linear defects (incomplete penetration, incomplete fusion, etc.), as shown in Figure 1. The traditional welding process generally carries on quality inspection after welding, which is easy to produce welding defects and affects the welding quality and production efficiency. Hull assembly is the prior process of welding process. High-precision assembly quality can improve welding quality and reduce welding time. Deformation and dimensional deviation are the main quality problems. As the assembly and welding hours account for more than 40% of the total working hours of ship construction, effective control plays a decisive role in improving the quality and efficiency of ship construction.

**Figure 2:**
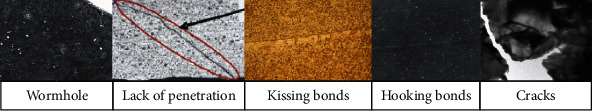
Scanning electron micrograph of internal welding defects.

Although the surface defects can be identified by manual inspection or camera, the defects inside the weld are not easy to identify. With the development of microtechnology, the nondestructive measurement of internal defects of weld can be achieved by using a scanning electron microscope and perspective electron microscope [33, 34]. The scanning electron micrograph of main internal defects is shown in Figure 2 [35]. Nondestructive testing (NDT) is the inspection on whether the weld quality meets the specified requirements without damaging the performance and integrity of the inspected weld, mainly methods including radiography, ultrasonic testing, eddy current, thermography, and liquid penetrant [36, 37]. NDT can provide rapid and economical methods for evaluating weld quality and has been widely used in various welding tests.

In the analysis of the assembly and welding process, the quality problems mainly are shown in Table 1. Among them, the welding factors include welding voltage, welding current, wire feeding speed, welding speed, bevel angle, weld not cleaned, welding material, and base metal thickness; the assembly factors include assembly sequence, margin, positioning, compensation, and spot welding parameters. Therefore, in order to effectively predict and control the process, it is necessary to complete the corresponding process decision through the process parameters and process data collection, data analysis, fusion, and quality prediction model.

## 3. Technical Process of Quality Prediction and Control

The traditional ship assembly and welding process quality control is used to compare the quality inspection results with the process requirements after welding to realize the product quality evaluation. In case of quality problems such as deformation, stress concentration and crack, postdeformation correction, and destress treatment shall be carried out. If cracks or other quality problems occur, the rejection rate will be increased and even the whole batch of products will be scrapped. Due to being time-consuming, high scrap rate, easy rework, low efficiency, high cost, and poor quality control, this method has been unable to meet the current requirements of the digital and intelligent construction of ship products. Therefore, this paper puts forward the quality prediction and control process of assembling and welding of ship group products based on the digital twin technology.

**Figure 3:**
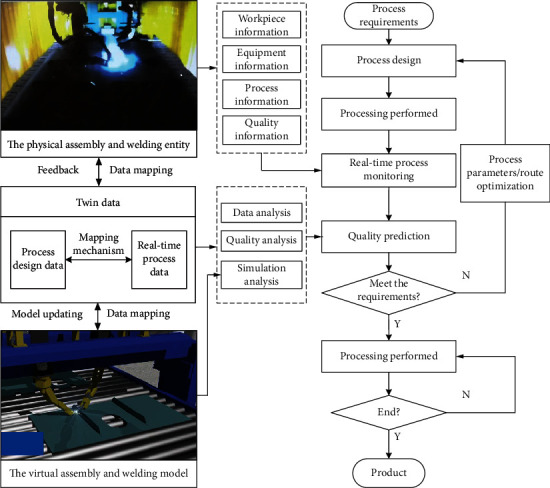
Quality prediction and control process of assembly and welding of ship group products based on digital twin.

As shown in Figure 3. Firstly, a virtual welding model corresponding to the physical assembly and welding entity is established to realize the high-fidelity mapping between the virtual model and the physical entity. Secondly, the quality data collection is realized by the sensors and data collection system arranged by physical entity. By combining the real-time process data, process design data, and process simulation data, the twin data of welding process were constructed, which realized the virtual model to simulate the welding process of physical entity simultaneously. Then, combined with historical data and real-time data, online prediction of process quality is realized by big data analysis. Auto regression model, support vector machine, artificial neural network, correlation vector machine, random forest, etc. are commonly used in big data analysis. In case of any abnormality, the correction machining parameters shall be timely carried out and fed back to the physical entity. Thus, the quality prediction and control of welding of ship group products are realized.

## 4. Establishment of Digital Twinning Model for Product Quality Prediction and Control

**Figure 4:**
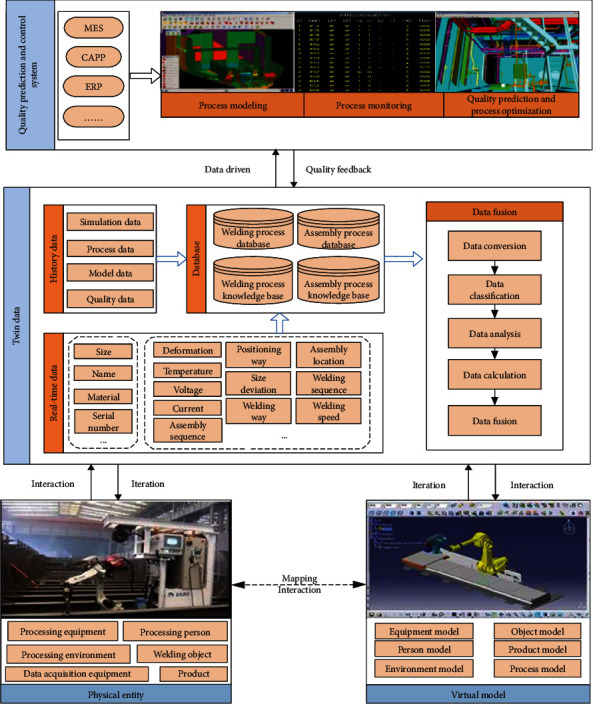
Digital twin model for prediction and control of mounting and welding quality of ship group products.

In order to meet the synchronous evolution of the virtual model and the physical perception data, a digital twin-based quality prediction and control model was constructed (as shown in Figure 4). Physical entity (PE), virtual model (VM), and quality prediction and control system (QS) are closely linked by digital twin data (DD). In the quality prediction and control system, the quality data in the physical entity can be collected in real time and stored as twin data; in the virtual model, the twin quality data stored in the quality prediction and control system can be analyzed and predicted, and the prediction results will be fed back to the physical entity for quality monitoring. The interaction and integration of the physical world and the information world of assembly and welding are realized. Define the quality of prediction and control model of ship group products based on digital twin:

(1) $$\begin{array}{c} W_{\text{DT}}=\text{PE}\cup \text{VM}\cup \text{DD}\cup \text{QS}, \end{array}$$

where PE refers to the physical assembly and welding entity, VM represents the virtual assembly and welding model, DD stands for the digital twin data, and QS is the quality prediction and control system.

PE is the set of all entities involved in the process. It is responsible for the execution of production activities. It also has the function of real-time collection and fusion of multisource heterogeneous data and makes dynamic responses according to process requirements, data monitoring, and process quality prediction results. The unified description of physical assembly and welding entity is as follows:

(2) $$\begin{array}{c} \text{PE}=\left[\text{pers},\text{proequis},\text{dcequis},\text{workps},\text{envi},\text{prods}\right]. \end{array}$$

pers is the processing personnel, proequis is the processing equipment, dcequis is the data acquisition equipment, workps is the welding object, envi is the processing environment, and prods is the product.

VM is a high-fidelity model corresponding to the physical entity, which can reflect the real-time state of the welding site and reproduce the physical assembly and welding entity. VM is mainly responsible for the simulation, prediction, evaluation, and optimization of the production process. The unified description of the virtual assembly and welding model is as follows:

(3) $$\begin{array}{c} \text{VM}=\left[\text{Mpers},\text{Mequis},\text{Mworkps},\text{Menvi},\text{Mprods},\text{Mpro}\right]. \end{array}$$

Mpers is the personnel twin model, Mequis is the equipment twin model, Mworkps is the welding object model, Menvi is the environment twin model, Mprods is the product model, and Mpro is the process twin model.

Digital twin data (DD) consists of real-time data and history data. The real-time data is the data generated in the current welding process, and the historical data is the previous relevant welding data. Twin data is the core of digital twin model, which provides data support for welding quality prediction and control. The twin data are uniformly described as

(4) $$\begin{array}{c} \text{DD}=\left[\text{cds},\text{hds}\right],\\ \text{cds}=\text{wpds}⊕\text{epids},\\ \text{hds}=\text{simds}⊕\text{prods}⊕\text{molds}⊕\text{quads}. \end{array}$$

cds is real-time data, including weldment data (wpds) and equipment data (epids); hds is history data, including the simulation data (simds), process data (prods), models data (molds), and quality data (quads).

QS is responsible for the monitoring and quality management and is the brain of the twin model. Firstly, the model of welding process is established to realize the synchronous evolution of virtual and real workshops. Secondly, intelligent sensing devices and systems are used to monitor the real-time state of welding manipulator, welding objects, operators, and environment. Finally, based on twin data, historical data, and machine learning algorithm to predict assembling and welding quality, the optimization scheme of parameters is provided to realize the prediction of loading and welding quality and dynamic adjustment of the process.

## 5. Real-Time Data Collection and Storage

A large amount of quality data will be generated in the process of assembly and welding, which will serve as an important basis for evaluating the quality of assembling and welding. Therefore, the collection and storage of data are very necessary.

### 5.1. Real-Time Data Collection

**Figure 5:**
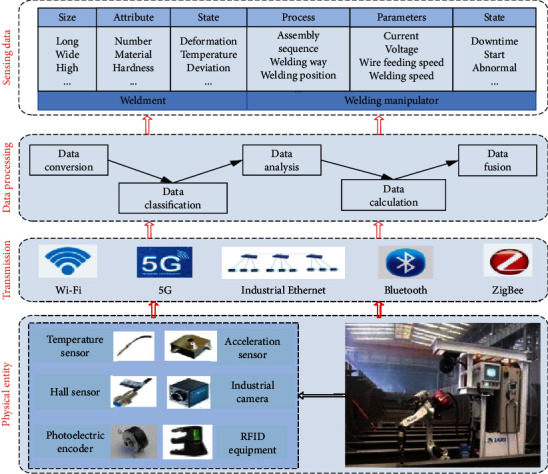
Real-time data acquisition of assembly and welding process.

As shown in Figure 5, the real-time data collection process is established. The physical sensor network and intelligent sensing equipment (system) to collect the assembly and welding equipment, welding parts, process, and quality information, such as a RFID device, is used to read the unique identification code of the welding parts and obtain the basic information of the welding parts, including the size, number, and process information of the welding parts. Hall sensor is used to detect welding current, welding voltage, and other data. Welding speed and wire feeding speed are detected by photoelectric code disk and speed sensor. An accelerometer and a gyroscope are used to detect the welding gun angle. The visual sensor is used to extract the information of the molten pool in the welding process, and the image processing technology is used to identify the features. On this basis, data transmission is carried out through a wireless network, 5G, Bluetooth, ZigBee, and industrial Internet, as well as data conversion, grouping, analysis, calculation, and fusion, and finally, valuable information oriented to process decision-making and quality prediction is formed.

### 5.2. Real-Time Data Storage

**Figure 6:**
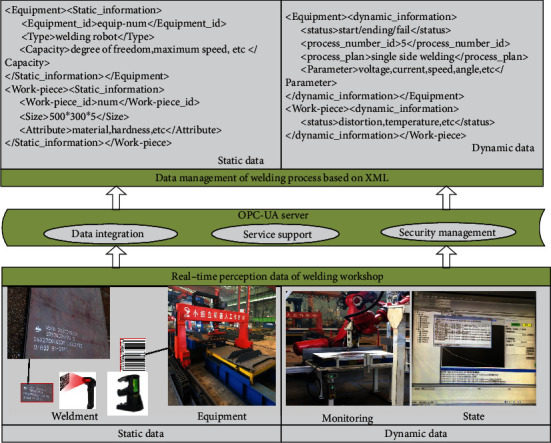
Data management and storage.

As shown in Figure 6, firstly, the real-time data of physical welding process is comprehensively analyzed, which is divided into dynamic data and static data. Static data refers to the information that will not change with the evolution of weldment in the process of weldment processing, such as equipment basic data and weldment basic data. The basic data of weldment includes weldment name, type, material, and process name; basic data of equipment includes equipment name, equipment model, and equipment processing capacity. Dynamic data refers to the information that will change accordingly as the processing state changes during the processing of weldment, such as the current process, start/completion information, and welding processing parameters of the product. The dynamic data directly reflects the real-time status and quality of the weldment and the operation status of the equipment. Secondly, according to the interface protocol between equipment/system and heterogeneous equipment of different manufacturers/models, the OPC-UA communication framework is used to realize the transmission of multisource heterogeneous data and uses XML text to manage and store real-time data.

## 6. Machine Learning-Based Ship Group Product Assembly and Welding Quality Prediction

With the rise of artificial intelligence, machine learning has developed rapidly. Machine learning is the use of algorithms to analyze data and learn from it and then make predictions and decisions on events. The machine learning algorithm is used to predict the assembly and welding quality online, which realizes the transformation from passive prevention to active prediction and control.

**Figure 7:**
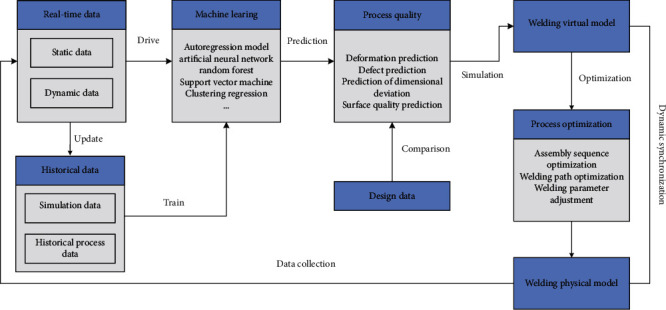
Prediction of assembly and welding quality based on machine learning.

As shown in Figure 7, real-time data (including dynamic data and static data) and historical data (process design data and simulation data) of the welding process are dynamically acquired by sensors and intelligent sensing devices to build quality twin data. The machine learning prediction model is trained by using the historical big data generated in the welding process. A large number of data are optimized interactively in the training process and packaged into corresponding prediction models. Through the real-time monitoring of digital twin and sensor data in the machining process, the condition monitoring and quality prediction of weldment can be realized. In the process of prediction, the real-time monitoring data can be tested and corrected according to the historical accumulated data. On the other hand, the historical data can be updated and expanded according to the real-time monitoring data. The welding model in the physical layer dynamically tracks and reflects the latest state of the weldment through its digital twin and optimizes the welding process through simulation, including assembly sequence optimization, welding path optimization, and welding parameter optimization. In this way, we can grasp the trend of quality change in advance, actively prevent quality problems, and achieve dynamic quality assurance. Finally, the fusion and intellectualization of physical information and virtual information in the welding process are realized.

## 7. Case Analysis

Taking the assembly and welding line of a shipyard as an example, it describes the realization process of the quality prediction and control method of assembly and welding based on digital twin. The production line consists of two assembly robots, two welding robots, and sensor systems (Hall sensor, optical code plate sensor, acceleration sensor, etc.). The weldment is composed of one panel and two webs, and the material is AH32. The panel size is 500 mm∗400 mm∗12 mm, and the web size is 400 mm∗100 mm∗10 mm. The chemical composition and mechanical properties are shown in Table 2.

The technological process includes the following: the assembly platform determines the assembly position through visual scanning; the assembly manipulator makes the floor plate and panel become positioned vertically; the welding manipulator realizes assembly positioning through spot welding; scanning is done again to determine the position and length of the welding seam, and then installation and welding are performed according to the welding process parameters and process flow imported by the system. During the welding process, the welding processing parameters (welding current, welding voltage, welding speed, etc.) are monitored, and the welding quality is predicted by artificial neural network.

### 7.1. Establishment of a Welding Process Model

**Figure 8:**
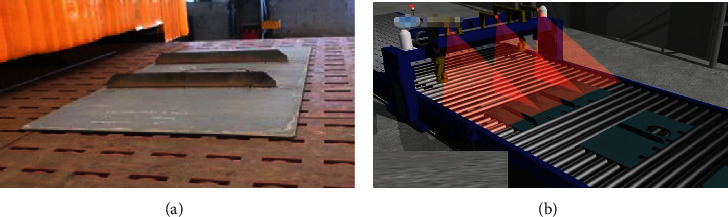
Welding process model: (a) product entity of the group; (b) simulation model of the production line.

The virtual model composed of welding parts and mechanical arm is established.  Figure 8(a) shows the product entity of the group, which is composed of a floor plate and two floor plates to complete the welding of the floor plate; Figure 8(b) shows the simulation model of the group production line.

### 7.2. Real-Time Data Collection and Storage

**Figure 9:**
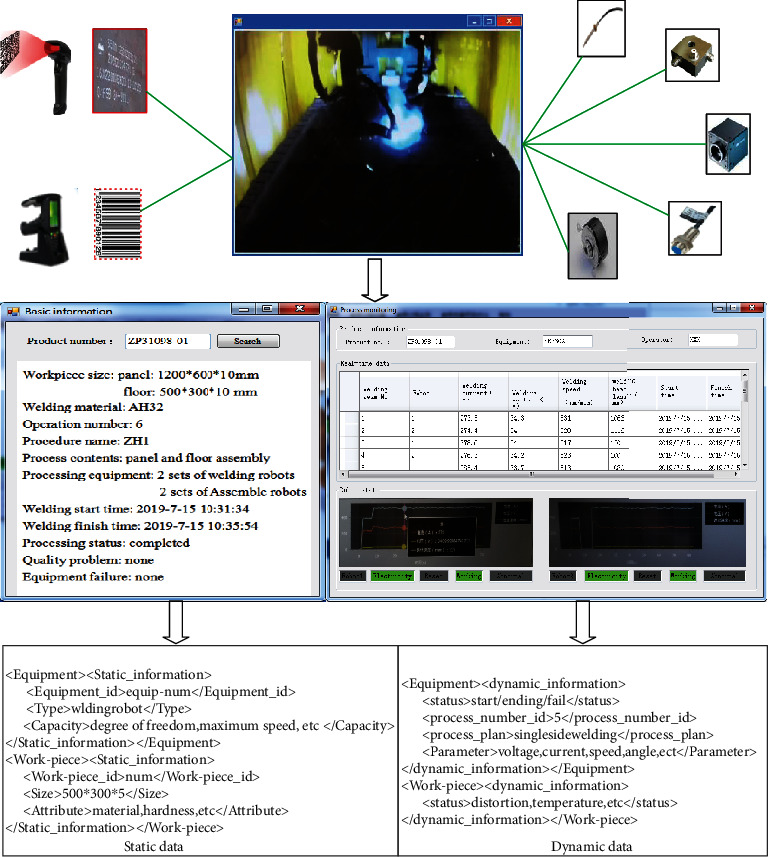
Data acquisition and monitoring of small group products.

According to the design requirements, real-time data of the assembly and welding process were collected to dynamically monitor the assembly and welding status of the products. As shown in Figure 9, specific implementation methods include (1) using RFID tags and bar codes to generate static information, such as the size of the welding parts, welding materials, process number, and equipment processing parameters; (2) obtain dynamic information based on multiple intelligent sensors (such as current sensor, speed sensor, temperature sensor, acceleration sensor, and industrial camera), such as equipment parameters, welding deformation, temperature field change, and process execution data; (3) establish data transmission network based on industrial Ethernet to ensure efficient data transmission and data collection; and (4) data storage and management and data related to manufacturing resource status/welding part status are stored in the database in XML format.

### 7.3. Quality Prediction of Welding Process Based on Artificial Neural Network

**Figure 10:**
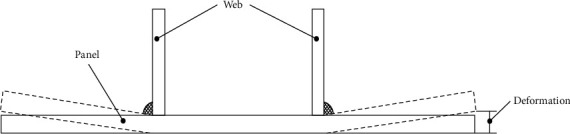
Weld angular distortion.

A BP neural network is a kind of back propagation neural network. It has strong nonlinear mapping ability, self-learning ability, and self-adaptive ability and has been widely used in nonlinear problems in material engineering [38–40]. Deformation is one of the main typical problems in welding. Welding deformation has a great impact on the installation accuracy of the structure, and excessive deformation will significantly reduce the bearing capacity of the structure. In this paper, the welding deformation of small group products is predicted by the BP neural network. Since the group product is a symmetrical structure, only the upward deformation of one side of the bottom plate is considered, as shown in Figure 10.

#### 7.3.1. BP Neural Network Model

**Figure 11:**
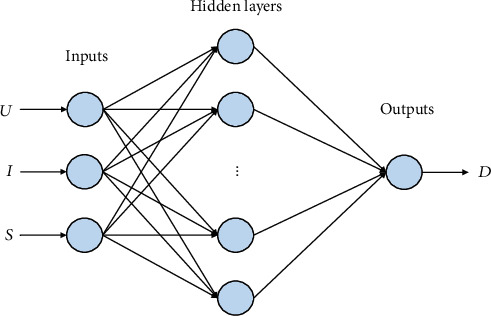
BP neural network prediction model.

Through the aforementioned analysis of welding deformation, three quality data of welding voltage (*U*), welding current (*I*), and welding speed (*S*) were selected as the input of the neural network, and the output was the deformation value (*D*). The learning rate was set at 0.1, the training target was set at 0.01, and the maximum training times was 100, to establish the welding deformation prediction model of the BP neural network. The network topology is “3-10-1,” as shown in Figure 11.

#### 7.3.2. Result Analysis

**Figure 12:**
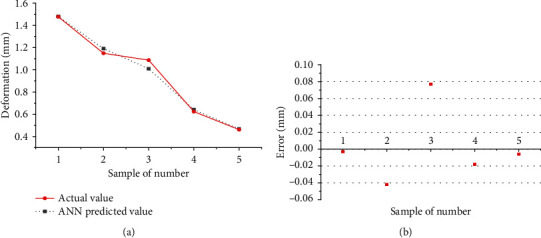
Experimental and ANN output: (a) data of deformation; (b) error.

As shown in Tables 3, 32 sets of data are selected as the basis for analysis. Twenty-seven data were used for training the network, and the remaining 5 data were used for testing the network. The result shows that the predicted value of the artificial neural network is consistent with the actual measured value, as shown in Figure 12. Comparing the predicted value with the actual value, it can be seen that the relative error is small, as shown in Table 4. Therefore, the established prediction model can predict the change trend of welding deformation according to the welding quality parameters and realize the active adjustment of the welding process.

## 8. Conclusions

The shipping industry is an important strategic industry related to national defense security and national economic development. With the continuous development of industrial Internet, big data, cloud computing, and other information technologies, ship building toward the direction of intelligence and automation has been accelerated. In this paper, digital twin technology is used to solve the problem of assembly and welding quality prediction and control of ship group products. First of all, real-time data acquisition and storage of assembly and welding process are used. Then, the welding quality prediction process based on machine learning is established. Finally, the BP neural network is used to establish the deformation prediction model of group products. The validity and accuracy of the model are verified by comparing the actual value with the predicted value. It can timely grasp the change trend of product processing quality and realize the active control of assembly and welding process quality.

Since welding quality is affected by many factors, it involves multiple data acquisition and fusion and multiobjective quality control, which will be the future research work.

## Figures and Tables

**Table 1:** The quality defects of ship group products.

Type of defect	Quality characteristic	Qualitative factors
Assembly defects	Deformation	(1) Positioning method; (2) assembly sequence; (3) welding parameters
	Size deviation	(1) Assembly margin; (2) assembly compensation amount; (3) positioning method
Surface welding defects	Porosity	(1) Welding current; (2) welding speed; (3) welding voltage; (4) the groove is not clean
	Slag inclusion	(1) Slag not cleaned; (2) groove form; (3) welding current; (4) welding speed
	Incomplete penetration	(1) Welding current
	Incomplete fusion	(1) Groove type; (2) assembly clearance; (3) wire feeding speed; (4) welding current; (5) electrode diameter
	Bite edge	(1) Welding current
	Welding tumor	(1) Welding voltage; (2) welding current; (3) wire feeding speed; (4) welding speed
	Welding pit	(1) Welding speed; (2) welding current; (3) welding voltage
Internal welding defects	Wormhole	(1) Welding speed; (2) welding current; (3) welding voltage; (4) welding material; (5) base metal thickness
	Lack of penetration	
	Kissing bonds	
	Hooking bonds	
	Cracks	

**Table 2:** Chemical composition and mechanical properties of the material.

C(%)	Mn(%)	Si(%)	S(%)	Yield strength (MPa)	Tensile strength (MPa)	Elongation (%)
≤0.18	0.70~1.60	0.10~0.50	≤0.04	315	440~590	22

**Table 3:** Historical data of welding deformation.

Experiment number	*U* (V)	*I* (A)	*S* (mm/s)	*D* (mm)
1	265	30	4	0.445
2	265	30	6	0.313
3	265	30	8	0.194
4	265	33	4	0.652
5	265	33	6	0.372
6	265	33	8	0.372
7	265	36	4	0.812
8	265	36	6	0.664
9	265	36	8	0.531
10	275	30	4	0.867
11	275	30	6	0.690
12	275	30	8	0.510
13	275	33	4	1.149
14	275	33	6	0.963
15	275	33	8	0.767
16	275	36	4	1.349
17	275	36	6	1.206
18	275	36	8	0.977
19	285	30	4	1.383
20	285	30	6	1.184
21	285	30	8	0.996
22	285	33	4	1.703
23	285	33	6	1.533
24	285	33	8	1.313
25	285	36	4	1.956
26	285	36	6	1.753
27	285	36	8	1.535
28	285	34	7	1.477
29	280	32	5	1.149
30	275	32	5	1.087
31	270	34	7	0.624
32	265	32	5	0.462

**Table 4:** Comparison of the predicted value and the actual value of welding deformation.

Experiment number	Actual value (mm)	Predicted value (mm)	Error (mm)
1	1.477	1.480	-0.003
2	1.149	1.191	-0.042
3	1.087	1.010	0.077
4	0.624	0.642	-0.018
5	0.462	0.468	-0.006

## Data Availability

All the data used to support the findings of this study are included within the article.
